# A Novel Therapeutic Strategy for the Treatment of Glioma, Combining Chemical and Molecular Targeting of Hsp90α

**DOI:** 10.3390/cancers3044228

**Published:** 2011-12-08

**Authors:** Adi Mehta, Leroy Shervington, Chinmay Munje, Amal Shervington

**Affiliations:** Brain Tumour North West, Faculty of Science and Technology, University of Central Lancashire, Preston, PR1 2HE, UK; E-Mails: abmehta@uclan.ac.uk (A.M.); lashervington@uclan.ac.uk (L.S.); crmunje@uclan.ac.uk (C.M.)

**Keywords:** sihsp90α, 17-AAG, glioblastoma, Hsp90α, Akt kinase, combinational treatment

## Abstract

Hsp90α's vital role in tumour survival and progression, together with its highly inducible expression profile in gliomas and its absence in normal tissue and cell lines validates it as a therapeutic target for glioma. Hsp90α was downregulated using the post-transcriptional RNAi strategy (sihsp90α) and a post-translational inhibitor, the benzoquinone antibiotic 17-AAG. Glioblastoma U87-MG and normal human astrocyte SVGp12 were treated with sihsp90α, 17-AAG and concurrent sihsp90α/17-AAG (combined treatment). Both Hsp90α gene silencing and the protein inhibitor approaches resulted in a dramatic reduction in cell viability. Results showed that sihsp90α, 17-AAG and a combination of sihsp90α/17-AAG, reduced cell viability by 27%, 75% and 88% (p < 0.001), respectively, after 72 h. *hsp90α* mRNA copy numbers were downregulated by 65%, 90% and 99% after 72 h treatment with sihsp90α, 17-AAG and sihsp90α/17-AAG, respectively. The relationship between Hsp90α protein expression and its client Akt kinase activity levels were monitored following treatment with sihsp90α, 17-AAG and sihsp90α/17-AAG. Akt kinase activity was downregulated as a direct consequence of Hsp90α inhibition. Both Hsp90α and Akt kinase levels were significantly downregulated after 72 h. Although, 17-AAG when used as a single agent reduces the Hsp90α protein and the Akt kinase levels, the efficacy demonstrated by combinatorial treatment was found to be far more effective. Combination treatment reduced the Hsp90α protein and Akt kinase levels to 4.3% and 43%, respectively, after 72 h. *hsp90α* mRNA expression detected in SVGp12 was negligible compared to U87-MG, also, the combination treatment did not compromise the normal cell viability. Taking into account the role of Hsp90α in tumour progression and the involvement of Akt kinase in cell signalling and the anti-apoptotic pathways in tumours, this double targets treatment infers a novel therapeutic strategy.

## 1. Introduction

Glioblastoma multiforme (GBM) is the most common malignant form of glioma, accounting for approximately 60–70% of all glioma cases and characterised by metastatic growth, malicious invasion and poor prognosis [1,2]. The current treatment for GBM consists of surgical removal of the tumour, followed by radiotherapy and concomitant use of the chemotherapeutic alkylating drug temozolomide (as recommended by The National Institute of Health and Clinical Excellence), which confers a survival period of 12–15 months [3]. Due to the modest effects of conventional treatment therapies, there is an urgent need for more effective ones.

The molecular chaperone heat shock protein 90 (Hsp90) has recently emerged as a vital target for cancer therapy. Hsp90 accounts for 1–2% of total protein in normal cells, which under stress conditions increases to 4–6% [4,5]. Hsp90 is upregulated in various human tumours where stress is prevalent, which may reflect the ability of malignant cells to maintain homeostasis in noxious environments [6,7]. Hsp90 binds to an array of client proteins, many of which are involved in apoptosis, cell survival and growth pathways [8]. Many of these client proteins are mutated or overexpressed in GBM [1] and therefore, inhibiting Hsp90 protein or its inducible component Hsp90α, could disrupt the oncogenic signalling pathways. Hsp90 silencing can be achieved using the benzoquinone antibiotic 17-allylamino-17-demethoxygeldanamycin (17-AAG) which is an Hsp90 inhibitor [9] and RNA interference (RNAi) using small interfering RNA (siRNA) [10].

17-AAG promotes growth inhibition in a number of cell lines, including gliomas, as well as antitumour activity *in vivo* and in preclinical models [11-15]. 17-AAG binds to the *N*-terminal domain of Hsp90 consequently inducing proteasomal degradation of its client proteins [16-18]. The combinatorial effect of 17-AAG on multiple signal transduction pathways involved in proliferation and survival, makes 17-AAG an ideal candidate for cancer therapy in GBM whose etiology is diverse. Moreover, the lipophilic nature of this drug allows easy access through blood brain barrier [1], and thus has a potential therapeutic value in GBM.

In humans, there are two major isoforms of Hsp90, namely Hsp90α and Hsp90β [19]. Although, Hsp90α levels in normal cells is lower compared to Hsp90β [19], Hsp90α expression is highly inducible to stressful stimuli such as heat shock, alcohol, heavy metals, oxidative stress and osmotic pressure changes, predominant in tumours [20]. In contrast, Hsp90β is thought to be constitutively expressed [21]. The high expression levels of Hsp90α has been associated with tumour progression, enhanced cell cycle regulation and induced cell signalling via tyrosine kinases [22]. A previous study in our laboratory showed high levels of both Hsp90α mRNA and protein expression in glioma cell lines and tissues in contrast to normal counterparts [23]. Therefore, silencing *hsp90α* can be a potential treatment strategy for GBM. The RNAi potential in gene therapy has been confirmed by several preclinical studies performed in the treatment of mammalian tumours [24-26]. siRNAs have emerged as an effective therapeutic strategy to silence disease genes, whereby it interferes with the translation of almost any mRNA [27]. Recently, we showed that three siRNA constructs target-specific to the human *hsp90α* gene significantly reduced *hsp90α* expression after 48 h [28]. Furthermore, the glioma cell lines treated with a combination of TMZ and siRNA, showed enhanced chemosensitivity to TMZ by a 13-fold reduction in the concentration of TMZ required in order to achieve the same cytotoxic effects as TMZ alone [28].

Clinical studies to date have shown only modest activity with molecular agents directed at single targets, due to coactivation of multiple tyrosine kinases and the presence of redundant signalling pathways [29]. Given the ability of 17-AAG to target several signalling pathways in GBM, we assessed its effects on tumour growth and survival, both as a single agent and in combination with siRNA (sihsp90α). This investigation aimed at downregulating Hsp90α mRNA and protein levels utilizing 17-AAG, siRNA and a combination of 17-AAG/sihsp90α. The efficacy and the ability of siRNA to synergise with 17-AAG and inhibit tumour growth was determined by measuring gene expression and protein levels. The Akt kinase protein activity, a client protein of Hsp90 widely known for its involvement in anti-apoptotic pathway [1], was also monitored.

## 2. Results and Discussion

### 2.1. Combinatorial Assays with 17-AAG and sihsp90α Inhibits Tumour Growth in U87-MG but Does not Affect SVGp12 Cell Viability

**Figure 1.**
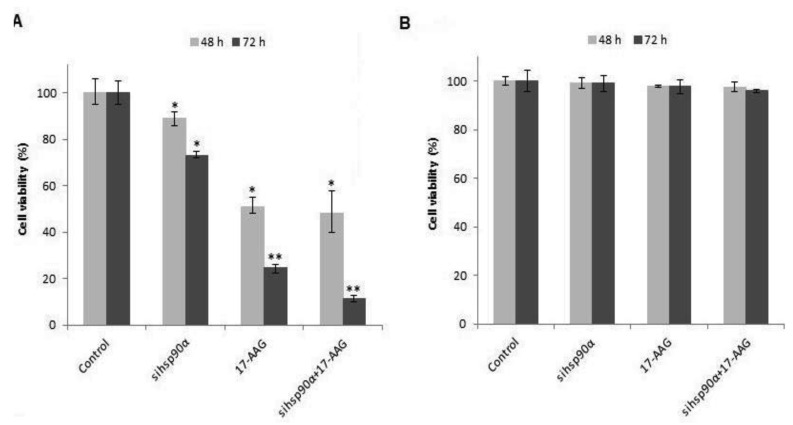
17-AAG and sihsp90α effect on cell viability *in vitro*. Cell viability was monitored for cells treated with 17-AAG and sihsp90α after 48 h and 72 h in (**A**) U87-MG and (**B**) SVGp12. Control represent untreated cells, sihsp90α represent cells treated with *hsp90α* specific siRNA. sihsp90α + 17-AAG represents concurrent assays where the cells were treated with 17-AAG and sihsp90α for either 48 h or 72 h. * p ≤ 0.05 and ** p ≤ 0.001 were considered statistically significant (Data values are mean ± SD, n = 3).

To determine the cell viability, U87-MG and SVGp12 cells were treated with 17-AAG and sihsp90α simultaneously with concurrent combinatorial assay. Both the gene silencing and protein inhibitor approaches showed dramatic reduction in cell viability in U87-MG (Figure 1 A). Data showed that sihsp90α, 17-AAG and combination of sihsp90α/17-AAG reduced cell viability by 27%, 75% and 88% after 72 h, respectively. Cytotoxic effects of 17-AAG, as a single agent, far exceeded the cytotoxic effects shown by sihsp90α at either 48 or 72 h. 17-AAG restricted tumour growth to 51% and 25% and sihsp90α treatment impaired tumour growth to 89% and 73% after 48 h and 72 h, respectively. To determine the therapeutic potential of this combination treatment, normal astrocyte cell line SVGp12 was treated with sihsp90α and 17-AAG for 48 h and 72 h (Figure 1B). Neither treatments significantly reduced SVGp12 cell viability after 48 h or 72 h thus demonstrating the tumour-specific targeting of this combination treatment.

### 2.2. sihsp90α Synergize with 17-AAG Treatment in U87-MG Cell Line

The ability of sihsp90α to synergize with 17-AAG in U87-MG was examined. The efficacy of the concurrent assay with 17-AAG and sihsp90α was assessed using interaction ratios generated by a ratio of the observed growth inhibition following treatment with both compounds *versus* growth inhibition using either compound independently. A ratio of 1 indicates additive growth inhibition, a ratio greater than 1 demonstrates synergistic growth inhibition, while a ratio less than 1 suggests subadditive effects on growth inhibition [1]. The interaction ratio calculated for concurrent treatments in this study is listed in Table 1. The results indicate that sihsp90α can synergize with 17-AAG and enhance treatment efficacy in glioblastoma. Upon statistical analysis, the concurrent treatment with 17-AAG and sihsp90α showed significant reduction in cell viability after 48 h and 72 h as compared to either treatment acting individually.

### 2.3. Silencing hsp90α in Vitro with sihsp90α

**Figure 2.**
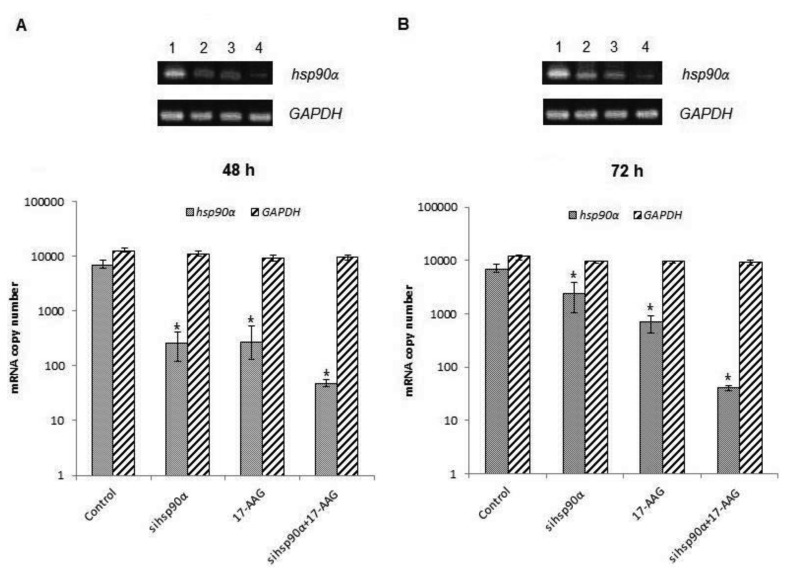
The effects of 17-AAG and sihsp90α on *hsp90α* expression. mRNA expression levels of *hsp90α* and *GAPDH* in U87-MG cells treated with Hsp90 inhibitor 17-AAG and/or sihsp90α which targets exon 5 on the *hsp90α* gene were assessed by agarose gel electrophoresis and qRT-PCR for mRNA gene expression copy numbers after (**A**) 48 h and (**B**) 72 h. Lane (1) control, (2) sihsp90α, (3) 17-AAG, (4) sihsp90α + 17-AAG. sihsp90α represents cells treated with *hsp90α* specific siRNA. sihsp90α + 17-AAG represent concurrent assays where the cells were treated with 17-AAG and *sihsp90α* for either 48 h or 72 h. * p ≤ 0.05 was considered statistically significant (Data values are mean ± SD, n = 3).

**Figure 3.**
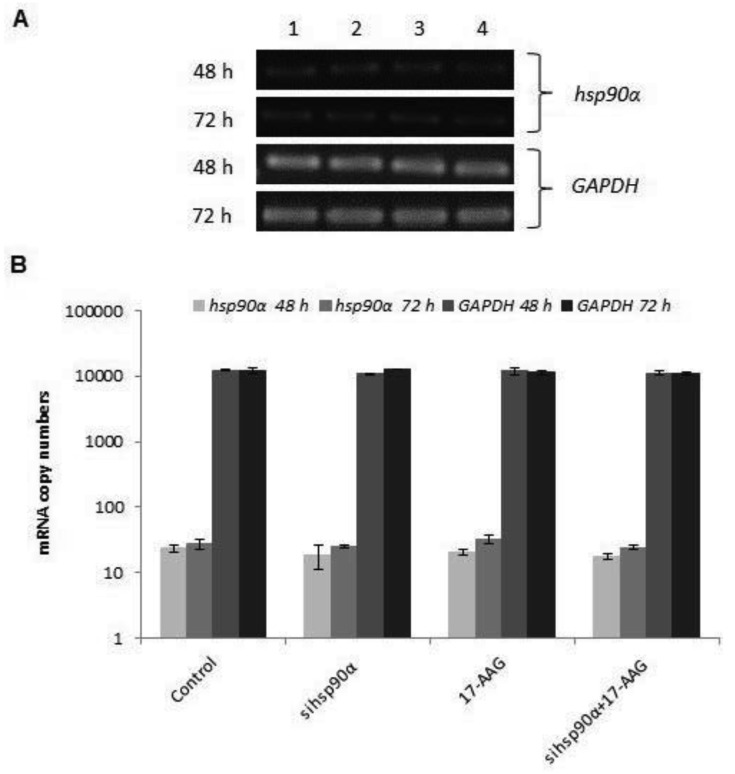
*hsp90α* transcription level in SVGp12. mRNA expression levels of *hsp90α* and *GAPDH* in SVGp12 cells treated with sihsp90α which targets exon 5 on the *hsp90α* gene and/or Hsp90 inhibitor 17-AAG were assessed by agarose gel electrophoresis (**A**) and qRT-PCR (**B**), for mRNA gene expression copy numbers after 48 h and 72 h. Lane (1) control; (2) sihsp90α; (3) 17-AAG; (4) sihsp90α + 17-AAG. sihsp90α represents cells treated with *hsp90α* specific siRNA. sihsp90α + 17-AAG represent concurrent assays where the cells were treated with 17-AAG and *sihsp90α* for either 48 h or 72 h. Data values are mean ± SD, n = 3.

In order to assess the efficiency of silencing *hsp90α,* U87-MG cells were treated with 17-AAG, sihsp90α or combination of sihsp90α/17-AAG to measure *hsp90α* and *GAPDH* mRNA expression using qRT-PCR. Cells transfected with sihsp90α alone reduced the mRNA copy number by 96% after 48 h, however, the mRNA copy number recovered to 65% after 72 h (Figure 2A,B). 17-AAG demonstrated a successful downregulation of *hsp90α* expression and reduced mRNA copy numbers by 92% and 90% after 48 h and 72 h, respectively. A combinatorial treatment with sihsp90α and 17-AAG together silenced *hsp90α* by 99% after 48 h and 72 h. The qRT-PCR results were also validated by agarose gel electrophoresis (Figure 2A,B). The level of *hsp90α* and *GAPDH* was also quantitated in a normal human astrocyte cell line SVGp12 to validate targeting *hsp90α,* a therapeutic candidate in this study (Figure 3A,B). The *hsp90α* levels in SVGp12 demonstrated negligible though detectable levels of mRNA. The different treatments did not have any significant effect on transcriptional regulation of *hsp90α.*

### 2.4. 17-AAG and sihsp90α Exposure Promotes Hsp90α Protein Degradation in U87-MG Cell Line

**Figure 4.**
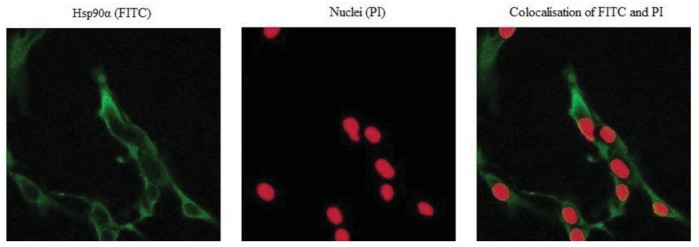
Hsp90α protein level in U87-MG assessed using immunohistochemistry. The cells were stained with FITC conjugate secondary antibody bound to Hsp90α antigen (green) and propidium iodide (PI) to detect nucleus (red) at ×40 objective magnifications. Hsp90α was mainly located in the cell cytoplasm.

Hsp90α protein levels were monitored by immunocytochemistry to correlate the transcription to the protein levels following sihsp90α and 17-AAG treatment. Figure 4 represents a sample of the stained cells showing a positive staining for Hsp90α. Hsp90α protein levels were significantly reduced following independent treatment with 17-AAG and sihsp90α. Most degradation of Hsp90α protein was observed after combinatorial treatment using 17-AAG and sihsp90α simultaneously, whereby only 4.3% of cells expressed Hsp90α after 48 h and 72 h (Table 2). When used as individual agents, sihsp90α and 17-AAG reduced Hsp90α protein levels to 30.9% and 11.4%, respectively. Based on the qRT-PCR and Hsp90α protein expression data, a clear correlation between the Hsp90α mRNA and protein expression levels was made.

### 2.5. Hsp90α Inhibition Promotes Degradation of Hsp90 Client Akt/PKB Kinase in U87-MG

**Figure 5.**
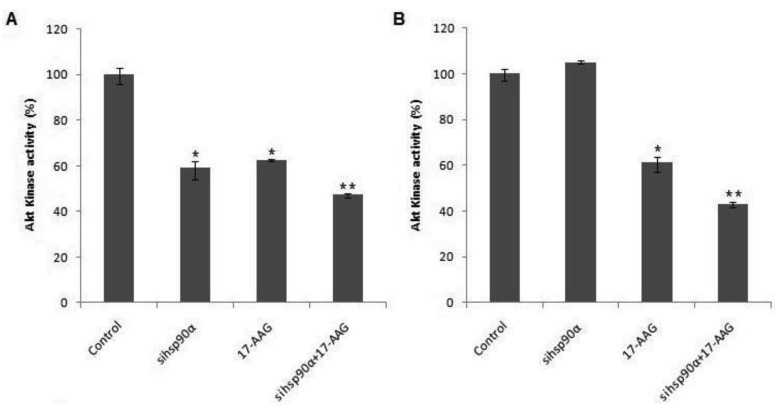
Hsp90α inhibition results in loss of Akt activity in U87-MG cell line. The Akt activity (%) was deduced as the fraction of the observed Akt activity in treated samples relative to the total Akt activity in the untreated sample after 48 h (**A**) and 72 h (**B**). sihsp90α represent cells treated with *hsp90α* specific siRNA. sihsp90α + 17-AAG represent concurrent assays where the cells were treated with sihsp90α and 17-AAG for either 48 h or 72 h. * p ≤ 0.05 and ** p ≤ 0.001 were considered statistically significant (Data values are mean ± SD, n = 3).

To examine whether Hsp90α inhibition was accompanied by rapid loss of Akt protein, the Akt kinase activity was monitored in control and treated U87-MG cells using a Akt/PKB kinase activity assay. A combination of sihsp90α and 17-AAG reduced Akt activity to 47.3% and 43% after 48 h and 72 h, respectively (Figure 5). When only 17-AAG was used, it was found to reduce Akt levels to 63% and 61 % after 48 h and 72 h treatments. Silencing *hsp90α* with siRNA inactivated Akt activity by 41% after 48 h. However, after 72 h the Akt activity was upregulated to over 100%. The expression profile of Akt emulates that of Hsp90α mRNA and the protein level following sihsp90α and 17-AAG treatments (Figure 2 and Table 2). The results of the qRT-PCR, Hsp90α protein and Akt activity indicates a strong correlation between mRNA, protein and Akt expression levels in U87-MG cell line (Table 3).

### 2.6. Discussion

In tumours, the induced Hsp90 expression is responsible for the addiction of tumour cells to multiple signalling pathways where Hsp90 clients play an oncogenic role [30]. Furthermore, the Hsp90 conformation in tumours has demonstrated a higher binding affinity for 17-AAG, compared to the native Hsp90 in normal cells [31]. Although, normal proteins have limited requirement for Hsp90 assistance, tumours are highly dependent on Hsp90 chaperone activity to cope with the lethal conditions prevalent in tumours and to regulate mutated and functionally deregulated proteins [32]. In the present study, the anti-tumour effect of 17-AAG and sihsp90α in U87-MG and not SVGp12 cells validates the reliance of brain tumours on Hsp90 for tumour growth and progression. In addition, the combinatorial therapy of 17-AAG and sihsp90α may have therapeutic potential for GBM, taking into consideration, the ability of 17-AAG to target-specifically bind to tumour cells at a low concentration compared to conventional therapeutic drugs and the increasing therapeutic potential of RNAi technique for gene therapy [28,33].

In order to assess quantitative gene silencing in GBM using sihsp90α and 17-AAG, *hsp90α* and *GAPDH* (control) mRNA expressions were measured using qRT-PCR. Results showed that a combination of 17-AAG and sihsp90α significantly reduced *hsp90α* copy numbers by 99% in GBM after 48 h and 72 h. Sequence specific interactions between the siRNA and target mRNA are essential for RNAi functionality, and the target site recognition relies on the molecular mechanism of RNA-RNA interactions [34]. Our data reveals a therapeutic potential of the siRNA used in this study for glioma therapy. To further support our findings, the cytotoxic effects of sihsp90α and 17-AAG on a normal human astrocyte cell line SVGp12 was investigated. The SVGp12 cell viability was not significantly affected showing less than 5% reduction in viable cells after 48 h and 72 h which clearly demonstrated selective tumour cell targeting over normal cells. These findings correlate with previous studies in our laboratory, which shows that *hsp90α* expression is highly induced in U87-MG, compared to normal counterpart [23]. Since *hsp90α* was transcribed at basal level in SVGp12 and other normal brain cell lines [23], there was no need to carry out further investigations to utilizing these cell lines.

The transcription of human *hsp90α* gene is regulated by the 5′ upstream promoter sequences bearing the heat shock elements (HSEs) known to regulate gene expression of *hsp90α* [19]. The heat shock factor 1 (HSF1) binds to the HSEs and marks the initiation of *hsp90α* gene transcription. Under normal conditions, HSF1 is bound to cytosolic Hsp90 and Hsp70 and hence avoids transcription of heat shock genes. However, under stress, or upon inhibition of Hsp90, the Hsp90 chaperone refolds partially denatured client proteins which liberates HSF1, consequently resulting in its translocation to the nucleus where it initiates transcription of *hsp90* gene [35]. This negative feedback mechanism may be a continued source of Hsp90 in tumour cells treated with 17-AAG or sihsp90α, which may be responsible for tumour survival even after 72 h. In this study, we targeted the inducible isoform *hsp90α* mRNA using sihsp90α and Hsp90 protein using 17-AAG with an aim to achieve maximum Hsp90 inhibition. The results from this study indicate that the enhanced synergistic cytotoxicity observed in glioblastoma U87-MG and not in normal astrocyte cell line SVGp12, using combination treatment with sihsp90α/ 17-AAG, may be due to a greater degree of Hsp90 inhibition. Our results also indicate that the normal astrocytes SVGp12 are not dependent on Hsp90 for cell growth and survival.

The fundamental role of Hsp90α in tumour invasion and cell cycle has previously been investigated [36]. However, altered *hsp90α* expression post 17-AAG treatment has not been studied in U87-MG cells. Nevertheless, a study by Clarke *et al.* investigating *hsp90α* gene expression in colon adenocarcinoma cell lines showed that the *hsp90α* expression in HCT116 (human colon cancer cells) following 17-AAG treatment was induced [37]. The same treatment on HT29 (human colon cancer grade II cell line) had no influence on *hsp90α* mRNA levels, suggesting that the post-transcriptional regulation of *hsp90α* after 17-AAG treatment was cell line dependent [37]. In GBM, 17-AAG was observed to downregulate *hsp90α* mRNA expression after 48 h and 72 h which correlates with its cytotoxic effects *in vitro*.

Hsp90 expression in tumours is 2 to 10-fold higher as compared to its basal expression in normal counterparts [38]. In addition to chaperoning mutated and deregulated client proteins, Hsp90 is of critical importance in tumour survival and progression. Theoretically, silencing *hsp90α* gene with sihsp90α should lead to tumour demise. The overall results from this study reveal non-complementarity between the cell viability and qRT-PCR data after sihsp90α treatment. Although, *hsp90α* mRNA expression was reduced by 96%, the cell viability was barely affected at 90%. *hsp90α* gene silencing was effective up to 48 h using sihsp90α, however, sihsp90α vulnerability to metabolism within the cell and its inability to duplicate [39], ultimately lead to *hsp90α* resurgence after 72 h. In contrast, the Hsp90 protein half-life of 36 h may suggest that the efficacy of *hsp90α* silencing on cell viability was modest after 48 h and 72 h, owing to the downstream regulation of anti-apoptotic clientele by Hsp90 [40].

The Hsp90α expression after treatment with 17-AAG and sihsp90α were measured by immunocytochemistry. The Hsp90α peptide was detected using FITC-conjugated secondary antibodies, taking into consideration the intensity, contrast and sharpness of the image. The Hsp90α protein expression profiles showed a distinct correlation with the *hsp90α* mRNA expression in U87-MG which strongly suggests that the regulation of Hsp90 occurs not only at post-translational level as proposed by previous observations [30], but also under transcriptional control.

The Hsp90 chaperone plays a vital role at maintaining Akt stability [40]. The involvement of Akt kinase in deregulated signalling pathways which facilitates tumour progression, survival and invasion, has been well documented [41]. Previous studies have demonstrated severe inhibition of Akt-dependent anti-apoptotic signalling pathways upon 17-AAG administration in gastric cancers, urinary bladder cancer and osteosarcoma [30,42,43]. To determine whether Hsp90α inhibition/silencing disturbed Akt levels in U87-MG, Akt kinase activity was measured using a Akt/PKB kinase activity kit. The present study showed downregulation of Akt kinase levels post 17-AAG and sihsp90α treatment which decisively contributes to the cytotoxic potencies of 17-AAG and sihsp90α after 48 h and 72 h. The decrease in cell viability observed in U87-MG cells may be attributed to the 17-AAG-dependent inhibition of Akt activity in addition to the sihsp90α-mediated *hsp90α* downregulation which counteracts the tumour survival response of *hsp90α* upregulation [22], eventually leading to Hsp90 depletion and apoptosis. It has been previously demonstrated that depletion of *hsp90β* by siRNA can induce apoptosis in multiple myeloma [44], which may suggest cooperating anti-apoptotic properties for Hsp90α and Hsp90β. In this study, downregulation of Hsp90α with sihsp90α was insufficient in suppressing Akt activity in U87-MG after 72 h, possibly due to the anti-apoptotic role of Hsp90β via association with Bcl-2 leading to caspase inactivation and to cell survival [45]. Also, it could be attributed to the inability of siRNA to duplicate, reducing silencing efficacy through cell division [39]. This may suggest why Akt levels recovered after 72 h following sihsp90α treatment.

Although, 17-AAG and sihsp90α triggered Akt degradation, interestingly, the most significant Akt suppression achieved was relatively higher than the corresponding Hsp90α levels. This may suggest that crucial components of the growth and survival signalling pathways, such as the Akt kinase, are not regulated by only Hsp90 chaperone. Indeed, upregulation of Hsp70 following Hsp90 inhibition using 17-AAG has been previously reported in prostate, cervical, and human colon tumours [37,46]. Furthermore, a recent proteomic study from our laboratory also showed the induced Hsp70 levels post 17-AAG exposure in glioblastoma cells (data unpublished). Thus, even though 17-AAG and sihsp90α inhibited Hsp90α levels in GBM, compensatory upregulation of Hsp70 may have proven to exhibit regulatory effects on Akt [47]. The Akt activity was reduced by 2.3-fold with the dual combination treatment after 72 h despite the Hsp70 compensation. This reduction of Akt activity demonstrates induced chemosensitivity in GBM since Hsp90 binds and protects Akt from dephosphorylation which is responsible for inactivation of Bcl-2 family protein Bad and caspase-9, known to be inducers of the intrinsic apoptosis pathway [20].

In our investigation, we have shown for the first time that a combination of 17-AAG and siRNA targeting *hsp90α* significantly suppresses GBM growth after 48 h and 72 h *in vitro,* clearly evident from the reduced Akt activity. This suggests that mutated proteins in GBM may have enhanced Hsp90α binding affinity and are more sensitive to Hsp90α inhibition [23]. Despite its anti-tumour activity in GBM, RNAi efficiency using siRNA is very limited *in vivo,* mainly due to siRNA degradation by nucleases, non-specific immune stimulation and poor cellular uptake [27,48,49]. However, intracellular delivery of biologically active siRNA has been successfully achieved using delivery systems [50,51]. The on-going work in our laboratory aims to address these limitations and to enhance cellular siRNA uptake, serum stability and pharmacokinetics using cell penetrating peptides.

## 3. Experimental Section

### 3.1. Cell Culture

The human brain tumour cell line U87-MG (glioblastoma multiforme) and human normal astrocyte cell line SVGp12 were purchased from European Collection of Cell Cultures (ECACC) and the American Type Culture Collection (ATCC), respectively. Both U87-MG and SVGp12 were cultured in Eagle's minimum essential medium (EMEM) (Lonza). The culture medium was supplemented with 2 mM L-glutamine (Sigma), 10% FBS (Gibco-BRL, Paisley, UK), 1% (v/v) non-essential amino acids and 1 mM sodium pyruvate. The cells were incubated at 37 °C in a humidified 5% CO2 incubator.

### 3.2. Drug Preparation and Treatment

**Figure 6.**
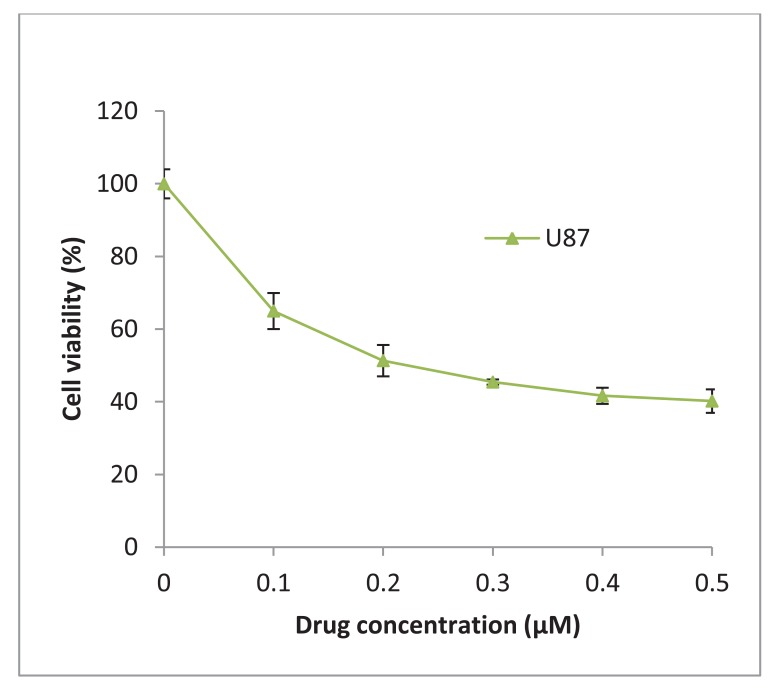
The IC_50_ for the Hsp90 inhibitor 17-AAG was assessed by increasing concentration of 17-AAG (0–0.5 μM). The data values are mean ± SD, n = 3.

17-AAG was obtained from InvivoGen (UK) and was reconstituted in DMSO to give a stock concentration of 2 mM. The glioblastoma U87-MG was treated with increasing concentrations of 17-AAG and a 50% inhibitory concentration (IC_50_) of 225 nM was determined (Figure 6). The stock solution was diluted in culture medium to achieve a final working concentration of 225 nM which was added to the cells and incubated for 48 h and/or 72 h.

### 3.3. sihsp90**α** Electroporation and Transfection

The *Silencer*^®^ Pre-designed siRNA used for transfections were obtained from Ambion (UK). The siRNA sequences consisted of 21-nt and targets Hsp90AA1 gene with sense strand: 5′ CGUGAUAAAGAAGUAAGCGtt 3′ (NM_005348.3) and antisense: 5′ CGCUUACUUCUUUAUC-ACGtt 3′. The annealed sihsp90α was resuspended in nuclease-free water to achieve a stock concentration of 100 μM which was further diluted to 50 μM working concentration prior to use.

The electroporation condition for sihsp90α used in this study has previously been optimised in our laboratory [28]. The siRNA transfections were carried out using the siPORT™ siRNA Electroporation Kit according to the manufacturer's instructions (Ambion, UK). Bio-Rad gene pulser Xcell was used for electroporation. Following transfection cells were transferred to a 25 cm^2^ tissue culture flask with pre-warmed medium and incubated for up to 72 h. For concurrent combinatorial assays involving sihsp90α and 17-AAG, 17-AAG (225 nM) was added to the pre-warmed medium prior to transferring cells into the flask.

### 3.4. Cell Viability Assay

The cell viability was assessed using CellTiter-Glo^®^ Luminescent cell viability assay (Promega, UK) [28]. Cells were seeded in a 96-well plate 24 h prior to treatment with sihsp90α, 17-AAG and/or combination of 17-AAG/sihsp90α. The luminescent signal was recorded using Tecan GENios Pro^®^ (Tecan, Austria).

### 3.5. mRNA Isolation, Reverse Transcription and qRT-PCR

An average of 1 ρg/cell of mRNA was extracted from cells using mRNA isolation kit (Roche Diagnostics, UK) according to manufacturer's instructions. Isolated mRNA (100 ng) was transcribed to cDNA using a First Strand cDNA Synthesis Kit (Roche Diagnostics, UK) which was used as a template for PCR. Quantitative Real Time PCR (qRT-PCR) was used to evaluate the expression of *hsp90α* and *GAPDH* (a housekeeping gene) in control and treated U87-MG cells. Primers were obtained from TIB MOLBIOL, Germany. The primer sequence and length of the amplicons were: *hsp90α* (189 bp) sense: 5′ tctggaagatccccagacac 3′, antisense: 5′ agtcatccctcagccagaga 3′, and *GAPDH* (238 bp) sense: 5′ gagtcaacggatttggtcgt 3′, antisense: 5′ ttgattttggagggatctcg 3′. qRT-PCR was performed in triplicate using Fast Start DNA master PLUS SYBR Green 1 (Roche Diagnostics, UK) in a LightCycler real-time PCR detection system (Roche Diagnostics, Germany) as described previously [23]. Quantitative amplification was monitored by the level of fluorescence reflecting the cycle number at the detection threshold (crossing point) using a standard curve [23].

### 3.6. Protein Extraction and Quantitative Assessment

Following treatment, the cells were collected and then lysed using CelLytic™ M Cell Lysis Reagent (Sigma, UK) supplemented with a protease inhibitor cocktail [52]. Total protein was isolated by centrifugation at 13,000 rpm for 15 min to remove cell debris. The protein concentration was determined using the Bradford protein assay.

### 3.7. Immunocytochemistry

Cells were seeded on culture slides in 6 well plates 24 h prior to treatment. Following treatment the cells were fixed with 4% paraformaldehyde, washed three times with warm PBS and subsequently permeablised with 0.3% Triton X-100 at room temperature for 7 min. The cells were washed three times with warm PBS and incubated in blocking solution followed by 1 h of incubating in primary monoclonal antibody against Hsp90α antigen (Cambridge Bioscience, UK). After incubating the cells, they were washed and exposed to a secondary antibody (goat anti-rat IgG FITC) for 1 h with gentle agitation. Finally, cells were washed and counter stained with PI (Vectashield, UK), mounted and fixed on slides for analysis. For each sample, a total of 150 cells were counted using Axiovert 200M LSM 510 laser scanning confocal microscope (Carl Zeiss Ltd, UK). Hsp90α was stained with FITC and the nucleus with propidium iodide (Figure 4).

### 3.8. Akt/PKB Kinase Assay

Akt/PKB kinase activity was measured using a non-radioactive Akt/PKB kinase activity assay that detects Akt/PKB kinase activity in the solution phase (Assay Designs, UK; Cat No. EKS-400A). Total protein isolated as described above and 10 μg of protein from each sample were used to perform Akt kinase assay according to the manufacturer's instructions.

### 3.9. Statistical Analysis

In this study, data was analysed using the PASW Statistics 18 package using the One-Sample Students T-test and Paired-Sample T-test. A p value of * p ≤ 0.05 and ** p ≤ 0.001 was considered as statistically significant.

## 4. Conclusions

We have demonstrated the anti-tumour effects in GBM utilizing 17-AAG and sihsp90α *in vitro*. The combinatorial treatment of 17-AAG/sihsp90α significantly downregulated Hsp90α mRNA and protein levels in GBM. Akt activity, a major survival kinase, was also found to be reduced. In contrast, neither of the treatments had a significant effect on SVGp12 cell viability. The transcriptional regulation of *hsp90α* was negligible in SVGp12. Given the role of Akt in growth signalling pathways, Hsp90-dependent Akt downregulation was accompanied by reduced cell viability. Thus, targeting Hsp90α using 17-AAG/sihsp90α may have a clinical prospect in treatment of GBM.

Given the role of Hsp70 and Hsp90β in cell survival pathways, future work could focus at downregulating Hsp70 and Hsp90β using protein inhibitors and siRNA respectively, and assessing its tumour inhibition potential which may enhance our understanding of the complex role played by the chaperones in tumour survival and progression.

## Figures and Tables

**Table 1.** sihsp90α synergizes with 17-AAG enhancing chemosensitivity in U87-MG. The efficacy of the concurrent assay with 17-AAG and sihsp90α was assessed using interaction ratios generated by a ratio of the observed growth inhibition following treatment with both compounds *versus* growth inhibition using either compound independently. The data values are mean ± SD, n = 3.

**Interaction Ratio**	**sihsp90α**	**17-AAG**

**after 48 h**	4.62 ± 1.35	1.05 ± 0.11
**after 72 h**	3.28 ± 0.19	1.18 ± 0.29

**Table 2.** Hsp90α expression in U87-MG post 17-AAG and sihsp90α treatment. The Hsp90α levels were deduced as the fraction of the observed Hsp90α levels in treated samples relative to the total Hsp90α levels in the untreated sample using immunofluorescence after 48 h and 72 h. Hsp90α expression analysis using immunofluorescence was performed by counting 150 cells. sihsp90α represent cells treated with *hsp90α* specific siRNA. sihsp90α + 17-AAG represent concurrent assays where the cells were treated with 17-AAG and sihsp90α for either 48 h or 72 h. This data is typical of three independent experiments. * p ≤ 0.05 and ** p ≤ 0.001 were considered statistically significant.

**% Cells Expressing Hsp90α after**	**Control**	**sihsp90α**	**17-AAG**	**sihsp90α + 17-AAG**

48 h	64.3 ± 4.2	30.9 ± 2.9 **	15.6 ± 1.4 **	4.7 ± 1.0 **
72 h	65.1 ± 4.6	54.5 ± 2.7 *	11.4 ± 1.2 **	4.3 ± 0.8 **

**Table 3.** The correlation profile between Hsp90α expression at mRNA and protein with Akt activity and cell viability in U87-MG cell line.

	**Hsp90α mRNA/100 ng Cells Extract (1 × 10^6^ Cells)**	**% Cells Expressing Hsp90α**	**% Akt/PKB Kinase Activity**	**% Cell Viability**
**Control**				
48 h	6,915 ± 863	64.3 ± 4.2	100 ± 3.5	100 ± 5
72 h	6,919 ± 867	65.1 ± 4.6	100 ± 2.5	100 ± 4.5
**siRNA**				
48 h	265 ± 95	30.9 ± 2.9	59.3 ± 3.8	89 ± 3
72 h	2,420 ± 967	54.5 ± 2.7	105.1 ± 0.79	73 ± 1.5
**17-AAG**				
48 h	269 ± 107	15.6 ± 1.4	62.7 ± 0.48	51 ± 3.5
72 h	694 ± 234	11.4 ± 1.2	61.1 ± 3.4	24.8 ± 1.9
**siRNA/17AAG**				
48 h	48 ± 7	4.7 ± 1.0	47.3 ± 0.91	48 ± 9
72 h	40 ± 4.5	4.3 ± 0.8	43 ± 1.28	11.3 ± 1.5
